# The gill transcriptome of threatened European freshwater mussels

**DOI:** 10.1038/s41597-022-01613-x

**Published:** 2022-08-13

**Authors:** André Gomes-dos-Santos, André M. Machado, L. Filipe C. Castro, Vincent Prié, Amílcar Teixeira, Manuel Lopes-Lima, Elsa Froufe

**Affiliations:** 1 CIIMAR/CIMAR — Interdisciplinary Centre of Marine and Environmental Research, University of Porto, Terminal de Cruzeiros do Porto de Leixões, Avenida General Norton de Matos, S/N, P 4450-208 Matosinhos, Portugal; 2 Department of Biology, Faculty of Sciences, University of Porto, Rua do Campo Alegre 1021/1055, 4169-007 Porto, Portugal; 3 National Museum of Natural History (MNHN), CNRS, SU, EPHE, UA CP 51, 57 rue Cuvier, 75005 Paris, France; 4 Centro de Investigação de Montanha (CIMO), Instituto Politécnico de Bragança, Bragança, Portugal; 5 CIBIO/InBIO - Research Center in Biodiversity and Genetic Resources, Universidade do Porto, Campus Agrário de Vairão, Rua Padre Armando Quintas, 4485-661 Vairão, Portugal; 6 IUCN SSC Mollusc Specialist Group, c/o IUCN, David Attenborough Building, Pembroke St., Cambridge, England

**Keywords:** Transcriptomics, Conservation biology

## Abstract

Genomic tools applied to non-model organisms are critical to design successful conservation strategies of particularly threatened groups. Freshwater mussels of the Unionida order are among the most vulnerable taxa and yet almost no genetic resources are available. Here, we present the gill transcriptomes of five European freshwater mussels with high conservation concern: *Margaritifera margaritifera*, *Unio crassus*, *Unio pictorum*, *Unio mancus* and *Unio delphinus*. The final assemblies, with N50 values ranging from 1069–1895 bp and total BUSCO scores above 90% (Eukaryote and Metazoan databases), were structurally and functionally annotated, and made available. The transcriptomes here produced represent a valuable resource for future studies on these species’ biology and ultimately guide their conservation.

## Abstract (summary)

**Table:** 

Measurement(s)	transcriptomics
Technology Type(s)	Illumina sequencing
Sample Characteristic - Organism	Margaritifera margaritifera • Unio crassus • Unio delphinus • Unio mancus • Unio pictorum
Sample Characteristic - Location	Europe

## Background & Summary

Ever since genomics approaches have been applied to non-model organisms, they have been recognized as fundamental tools to study biodiversity and guide conservation actions, coining the term conservation genomics^1–4^. Genomic data provides a comprehensive and accurate framework enhancing the characterization of genetic variation, population structure and dynamics, selective pressures and adaptative traits that ultimately guide and prioritize applied conservation efforts^1–4^. Furthermore, genomic data are fundamental to construct predictive models to access the impact of human-mediated threats, such as biological invasions, resource depletion, and climate change^1,3,5^.

Freshwater mussels (Order Unionida) are molluscs extremely important to freshwater ecosystems where they play key ecological roles, such as nutrient and energy cycling and retention^6–8^. They also provide important direct (e.g., as food, pearls, and other raw materials) and indirect (e.g., water clearance, sediment mixing) services to humans^6,7,9^. These organisms are among the most threatened worldwide, with many species near extinction^10–12^. Of the thousand known species, only four whole genomes^13–16^ and less than 20 transcriptomes are available^17–29^. Of these, only one is from the European continent^23^. Here, we produce reference transcriptomes of five European species as baseline tools to support future studies. Genomic tools, such as transcriptomes, are key resources to study evolutionary and adaptive traits. Examples include, in the case of freshwater mussels, the unique obligatory parasitic interaction with a freshwater fish host (and occasionally other vertebrates), essential to disperse their larvae and complete the life cycle or the response to human-mediated threats, including climate change and habitat degradation^8,10^. Moreover, these species are ecological indicators, and the transcriptomes provide a catalogue of key genes and pathways, related to important stressors (e.g., temperature, oxygen availability), as well as basic mechanisms underlying freshwater mussel’s stress adaptation^17,19,23,24,30,31^.

**Fig. 1:**
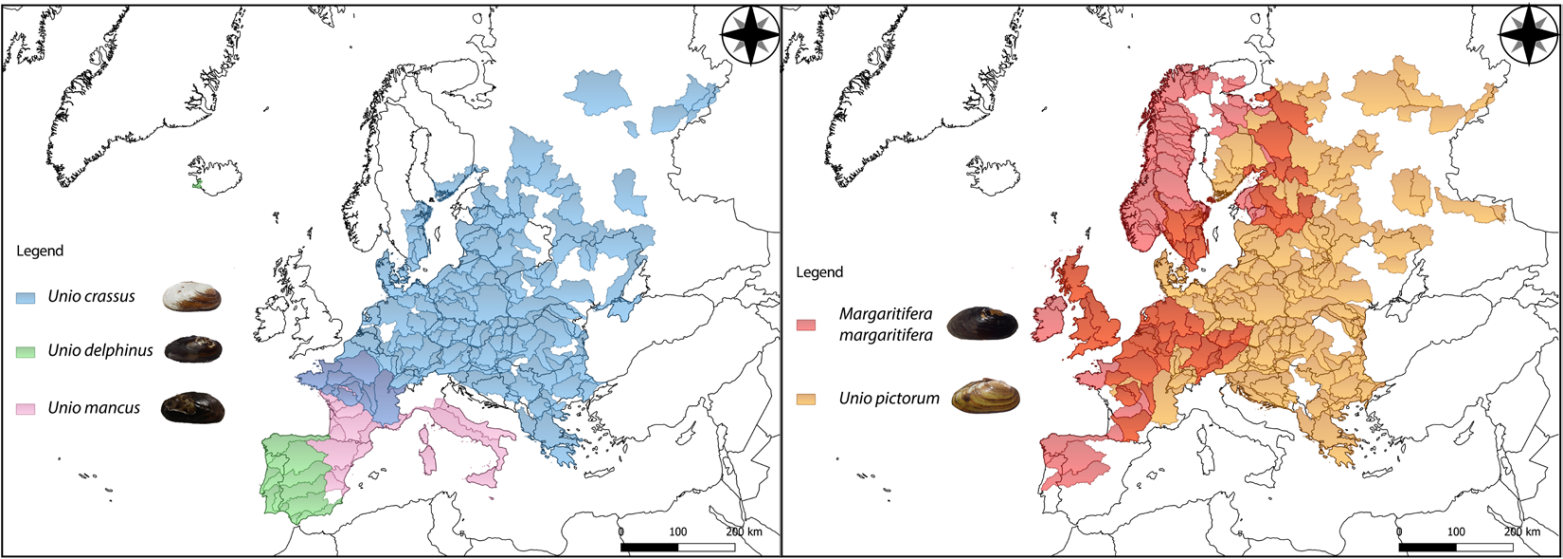
Maps of the five species’ potential distributions produced by overlapping points of recent presence records (obtained from Lopes-Lima *et al*.^10^) with the Hydrobasin level 5 polygons^59^. Overlapping distribution polygons between *Unio mancus* and *Unio crassus* are represented by a light purple shade, in the left panel. Overlapping distribution polygons between *Unio pictorum* and *Margaritifera margaritifera* are represented by an orange shade, in the right panel.

We present the gill transcriptome of the most emblematic freshwater pearl mussel, *Margaritifera margaritifera* (Linnaeus, 1758). This species was famous as a source of pearls throughout the last two millennia^13^. Currently, is among the most threatened freshwater mussel species in Europe, with many populations suffering massive declines, with up to 90% of European populations depleted by the 90 s, which is reflected in the current scattered distribution^32^ (Fig. 1). Recently, a whole-genome assembly was published^13^, adding to unique transcriptomic dataset of a very specialized tissue (i.e., kidney^23^). The current species conservation status is Endangered by the IUCN and is also listed in the EC Habitats Directive^33^. The other four transcriptomes are from the *Unio* genus, the type genus of the order Unionida, i.e., *Unio delphinus* Spengler, 1793, *Unio crassus* Philipsson in Retzius, 1788, *Unio pictorum* (Linnaeus, 1758) and *Unio mancus* Lamarck, 1819, for which no genomic resources have been produced at all. Two of these species, i.e., *U. crassus* and *U. pictorum*, although widely distributed (Fig. 1), have also suffered recent declines, with *U. crassus*, once considered the most abundant unionid in Europe, now listed as Endangered by the IUCN and also listed in the EC Habitats Directive^34^. The other two species have much more restricted distributions (Fig. 1), both suffering strong population losses, with *U. delphinus* listed as Near Threatened and *U. mancus* as Endangered by the IUCN^35,36^. The depleted conservative state of Unionida mussels is a global concern, being the second group with the highest percentage of threatened species (43%) and the group with the highest number of wild extinct species (6.3%)^37^.

In this context, increasing the genomic resources available for freshwater mussels, particularly of European species, is vital. The transcriptomes produced here offer a unique opportunity to explore and decipher the capability of these species to cope with current and future threats and ultimately guide conservation genomic studies to protect this highly threatened group of organisms.

## Methods

### Animal sampling

One individual of *M. margaritifera* was collected from the Tuela River in Portugal, one *U. crassus*, and one *U. pictorum* from the Dobra River in Croatia, one *U. mancus* from the Taravu River in France and one *U. delphinus* from the Rabaçal River in Portugal (Table 1), all adult individuals. Differentiated tissues were promptly flash frozen and stored at −80 °C, at CIIMAR tissue and mussels’ collection, as well as their respective shells.

**Table 1:** MixS descriptors for the five freshwater mussel species.

Sample	*Margaritifera margaritifera*	*Unio crassus*	*Unio pictorum*	*Unio mancus*	*Unio delphinus*
Investigation_type	Eukaryote	Eukaryote	Eukaryote	Eukaryote	Eukaryote
Project_name	Gill transcriptome of five freshwater musssles’ european species				
Lat_lon	41.862414; −6.931596	45.515500; 15.473240	45.515500; 15.473240	41.710606; 8.828512	41.564361; −7.258665
Geo_loc_name	Portugal	Croatia	Croatia	France	North of Portugal
Collection_date	7/6/2021	7/12/2019	7/12/2019	4/21/2021	3/20/2021
Env_package	Water	Water	Water	Water	Water
Seq_meth	Illumina HiSeq 4000	Illumina HiSeq 4000	Illumina HiSeq 4000	Illumina HiSeq 4000	Illumina HiSeq 4000
Assembly method	Trinity	Trinity	Trinity	Trinity	Trinity
Collector	Amilcar Teixeira	Manuel Lopes-Lima	Manuel Lopes-Lima	Vincent Prié	Amilcar Teixeira
Sex	Undetermined	Undetermined	Undetermined	Undetermined	Undetermined
Maturity	Mature	Mature	Mature	Mature	Mature

### RNA extraction, library construction, and sequencing

Total RNA of gills was extracted using the NZY Total RNA Isolation kit (NZYTech, Lda. - Genes and Enzymes), following the manufacturer’s instructions. RNA concentration (ng/μl) and quality measurement (OD260/280 ratio values) were obtained using a DS-11 Series Spectrophotometer/Fluorometer (*M. margaritifera* - 380.75 ng/μl, *U. crassus* – 478.290 ng/μl, *U. pictorum* - 375.461 ng/μl, *U. mancus* - 225.815 ng/μl, *U. delphinus* – 230.234 ng/μl). The extracted total RNA from the five samples was sent to Macrogen, Inc to build strand-specific libraries, with an insert size of 250–300 bp and sequenced using 150 bp paired-end reads on the Illumina HiSeq 4000 platform.

### Pre-assembly processing

**Fig. 2:**
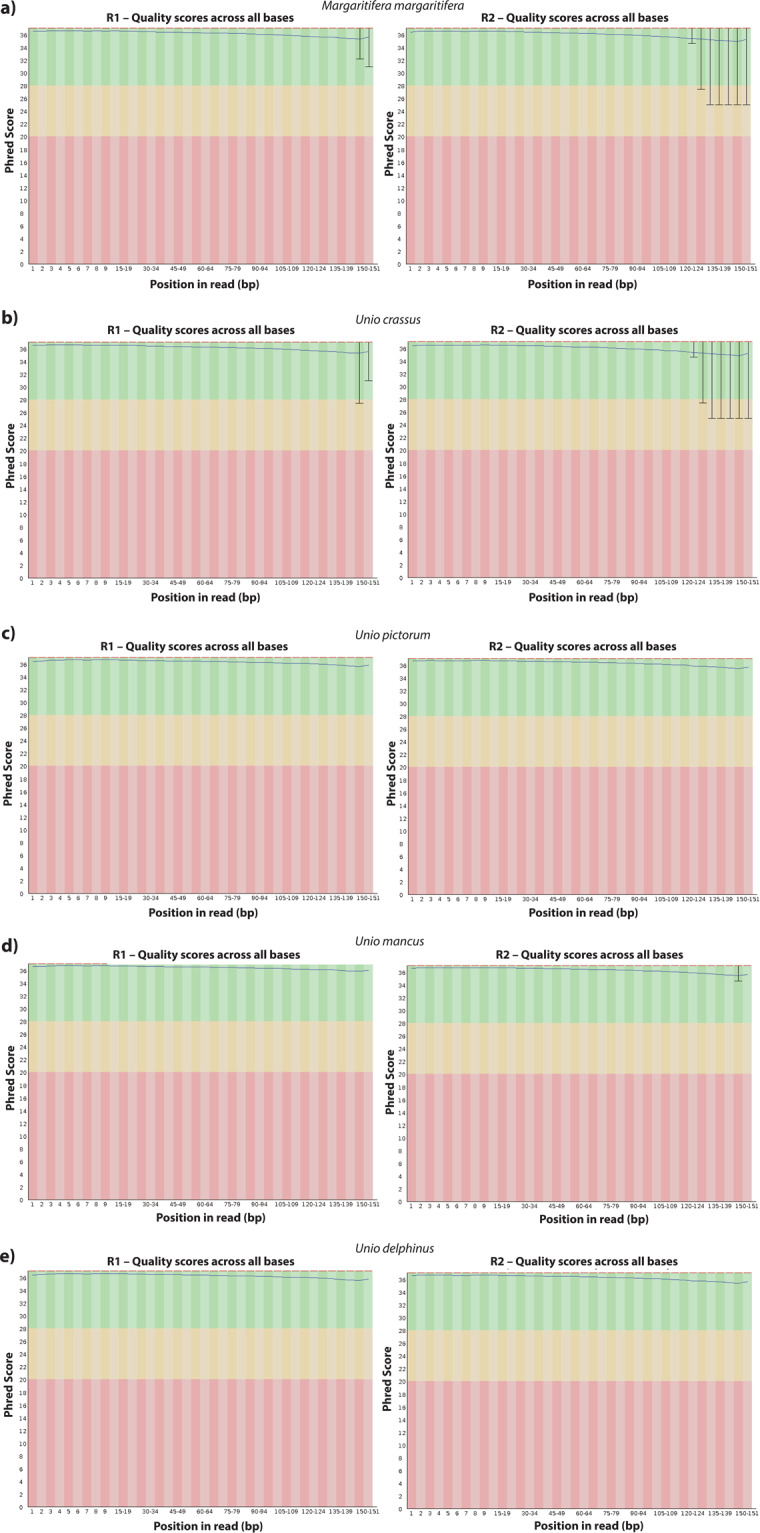
Bioinformatics pipeline applied for the transcriptome assembly and annotation. Auxiliary representative figures were created with BioRender.com.

Raw reads datasets for each sample were first inspected with FastQC (version 0.11.8) software (http://www.bioinformatics.babraham.ac.uk/projects/fastqc/). Afterwards, reads were quality-filter and Illumina adaptors were removed using Trimmomatic (version 0.38)^38^, using the parameters LEADING:5 TRAILING:5 SLIDINGWINDOW:5:20 MINLEN:36 (Fig. 2). Trimmed reads were correct for random sequencing errors using a kmer-based error correction approach in Rcorrector (version 1.0.3)^39^ with default parameters and after imported to Centrifuge (version 1.0.3-beta)^40^ to taxonomically classify them using a pre-compiled nucleotide database from NCBI (ftp://ftp.ccb.jhu.edu/pub/infphilo/centrifuge/data/) (version nt_2018_3_3). All reads whose classification did not belong to the Mollusca superclass (Taxon Id: 6447) were removed (Fig. 2).

### *De novo* transcriptome assembly

The fully processed reads were used for the whole transcriptome *de novo* assembly for each sample, with Trinity (version 2.13.2)^41,42^ using the default parameters. To ensure the removal of contamination, the assembled transcripts were blasted against nucleotide database of NCBI (NCBI-nt; (Download; 24/08/2021)^43^) and Univec (Download; 02/04/2019) databases using Blast-n (version 2.11.0)^44^ (Fig. 2). Afterwards, transcripts that held a minimum alignment length of 100 bp, an e-value cut-off of 1e-5, identity score of 90%, and a match to Mollusca phylum (NCBI: taxid 6447) or without matches at all, were retained. On the other hand, transcripts matching other taxa in the NCBI-nt database or any match to the Univec database were considered contaminants and removed from the datasets.

### Redundancy removal

Before proceeding to open reading frame (ORF) prediction, transcript redundancy was removed using a hierarchical contig clustering approach, implemented with Corset (version 1.0.9)^45^. For that, raw reads for each sample were mapped onto their respective transcriptome assemblies using Bowtie2 (version 2.3.5) (parameter:–no-mixed–no-discordant–end-to-end–all–score-min L,− 0.1,− 0.1). After Corset (version 1.0.9)^45^ was used to cluster contigs, filtered redundancies, and exclude any transcripts containing less than 10 mapped reads. The overall quality of the five transcriptomes (before and after redundancy removal) was assessed for completeness, using Benchmarking Universal Single-Copy Orthologs tool (BUSCO version 3.0.2) with the lineage-specific libraries for Eukaryota and Metazoa^46^ and for structural integrity using TransRate (version 1.0.3)^47^ (Fig. 2).

### Open reading frame prediction and transcriptome annotation

The open reading frames (ORFs) for each non-redundant transcriptome, were produced using Transdecoder (version 5.3.0) (https://transdecoder.github.io/) (Fig. 2). During the ORF prediction process, the homology and protein searches were performed in UniProtKB/Swiss-Prot^48^ and PFAM databases^49^ using the Blast-p (version 2.12.0)^44^ and hmmscan of hmmer2 package (version 2.4i)^50^ software, respectively. Next, the Gtf/Gff Analysis Toolkit (AGAT) (version 0.8.0)^51^ was applied to produce the structural annotation file (in gff3 format) from the Transdecoder output file (.gff) and transcriptome assembly file (.fasta). In the end, the AGAT tool was used to extract the protein and transcript fasta files with the names properly uniformized and formatted per species. Afterwards, the functional annotation was performed with InterProScan tool (version 5.44.80) and Blast-n/p/x searches in several databases. While the proteins per species were queried against InterPro (Download; 30/03/2019) and protein databases of NCBI (NCBI-RefSeq – Reference Sequence Database (Download; 10/03/2022)^52^ NCBI-nr – non-redundant database of NCBI (Download; 15/12/2021)^43^ with the Blast-p/x tool of DIAMOND software (version version 2.0.13)^53^, the transcripts were searched by Blast-n/x in NCBI-nt and NCBI-nr databases, with Blast-n tool of NCBI and Blast-x tool of DIAMOND software. In the end, all blast (outfmt6 files) and InterProScan (tsv file) outputs were integrated into the gff3 annotation file with the AGAT tool. The putative gene name per sequence was assigned based on the best blast hit (Gene symbol – NCBI Accession Number) and following the ranking: 1- Blast-p Hit in RefSeq database; 2 - Blast-p Hit in NCBI-nr database; 3 - Blast-x Hit in NCBI-nr database; 4 - Blast-n Hit in NCBI-nt database.

## Data Records

The raw reads for each sample were deposited at the NCBI Sequence Read Archive with the accessions numbers: SRR19261768 (MM), SRR19261764 (UD), SRR19261767 (UP), SRR19261765 (UM), SRR19261766 (UC)^54^; the BioSample accessions numbers: SAMN28495338 (MM), SAMN28495283 (UD), SAMN28495235 (UP), SAMN28495263 (UM), SAMN28495214 (UC) and under BioProject PRJNA839062^55^. The remaining information was uploaded to figshare^56^. In detailed, the files uploaded to figshare include, the filtered trinity redundant assemblies (_trinity_filtered.fasta), the non-redundant transcriptomes (_transcriptome.fa), transcripts files (_genes.fa), messenger RNA file (_mrna.fa), open reading frames predictions (_cds.fa), open reading frames proteins predictions (_proteins.fa) as well as the annotation files (_annotation_sorted.gff3.gz).

## Technical Validation

### Raw datasets and pre-assembly processing quality control

**Fig. 3:**
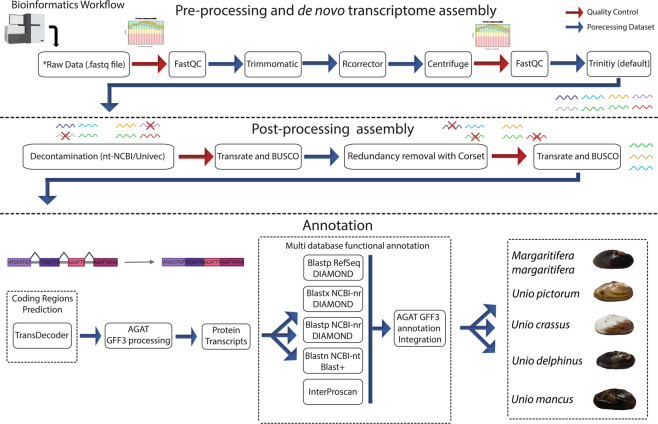
FastQC quality report of the trimmed and decontaminated RNA-seq reads (after Centrifuge for each species. (**a**) *Margaritifera margaritifera*; (**b**) *Unio crassus;* (**c**) *Unio pictorum*; (**d**) *Unio mancus*; and (**e**) *Unio delphinus*.

The raw sequencing outputs resulted in a total of 131051306 million reads (M) for *M. margaritifera*, 132002266 M for *U. crassus*, 104108396 M for *U. pictorum*, 100704688 M for *U. mancus*, and 112439686 M for *U. delphinus*. Although the initial overall quality of raw data was considerably good (Fig. 3), the datasets were further improved by quality trimming (Trimmomatic), error-correction (Rcorrector), and decontaminated (Centrifuge) (Fig. 3). The number of reads removed during the pre-assembly processing represented less than 3% of each dataset (Table 2) and the overall Phred scores were all above 25 (Fig. 3a–e).

**Table 2:** Basic statistics of raw sequencing datasets and percentages of removed reads at each step of the preassembly processing strategy.

Basic Statistics	Total Transcriptome	Non redundant Transcriptome	Total Transcriptome	Non redundant Transcriptome	Total Transcriptome	Non redundant Transcriptome	Total Transcriptome	Non redundant Transcriptome	Total Transcriptome	Non redundant Transcriptome	
	*Margaritifera margaritifera*	*Margaritifera margaritifera*	*Unio crassus*	*Unio crassus*	*Unio pictorum*	*Unio pictorum*	*Unio mancus*	*Unio mancus*	*Unio delphinus*	*Unio delphinus*	
Number of transcripts	1694677	470852	1304611	169668	232124	68670	234695	65620	280001	82542	
n bases	1052464277	442302372	1002862692	262637793	189129150	83762650	198791465	89666570	224567067	103248722	
Mean transcript lenght (bp)	621.02389	939.36603	768.67926	1547.94894	814.75652	1219.7852	847.00815	1366.44881	802.01073	1250.86286	
Number of transcripts over 1 K nt	214128	134690	235872	104192	53293	28701	54754	31276	62078	35904	27.78417362
Number of transcripts over 10 K	1189	261	1905	453	7	5	33	15	24	12	
N90 trancript lenght (bp)	284	499	313	816	314	582	322	659	309	612	
N70 trancript lenght (bp)	462	759	589	1324	697	1037	732	1168	677	1047	
N50 trancript lenght (bp)	773	1069	1187	1889	1447	1688	1569	1895	1400	1669	
N30 trancript lenght (bp)	1475	1619	2409	2864	2438	2589	2635	2870	2426	2600	
N10 trancript lenght (bp)	3783	3281	5504	5458	4073	4174	4427	4592	4108	4252	
Percentage of GC (%)	0.36365	0.35712	0.35352	0.34896	0.35511	0.35179	0.35899	0.35468	0.36814	0.36893	
**Busco analysis (%)**											
BUSCO Complete (Single + Duplicated)	93.7/94.5	85.8/89.4	97.1/98.1	92.1/93.1	87.5/83.1	83.8/79.7	89.8/88.2	85.2/83.9	92.1/88.3	89.1/84.8	
BUSCO Single*	45.5/47.4	83.8/85.8	44.6/43.6	90.8/90.5	58.1/57.8	80.5/77.8	62.7/64.6	82.2/82.7	62.7/64.0	81.2/80.8	
BUSCO Duplicated*	48.2/47.1	2.0/3.6	52.5/54.5	1.3/2.6	29.4/25.3	3.3/1.9	27.1/23.6	3.0/1.2	29.4/24.3	7.9/4.0	
BUSCO Fragmented*	4.0/4.5	8.3/6.1	2.3/1.6	3.6/3.9	7.9/10.2	6.9/7.4	6.6/8.0	7.6/6.4	5.6/7.8	5.0/6.1	
BUSCO Missing*	2.3/1.0	5.9/4.5	0.6/0.3	4.3/3.0	4.6/6.7	9.3/12.9	3.6/3.8	7.2/9.7	2.3/3.9	5.9/9.1	
Total Buscos Found*	97.7/99.0	94.1/95.5	99.4/99.7	95.7/97.0	95.4/93.3	90.7/87.1	96.4/96.8	92.8/90.3	97.7/96.1	94.1/90.4	

### Transcriptome assembly metrics

The *de novo* transcriptome assemblies were performed using Trinity, with default paraments, which has been successfully applied for other Unionida transcriptome assembly projects^17,20–23^. Furthermore, the overall completeness of the transcriptome assemblies was evaluated using Benchmarking Universal Single-Copy Orthologs (BUSCO), by searching the Eukaryota (n:303) and Metazoa (n:978) near-universal single-copy orthologs databases, for all species. The overall metrics for each transcriptome *de novo* assembly, as well as their corresponding BUSCO scores, are presented in Table 3. The general assembly metrics of *U. pictorum*, *U. mancus*, and *U. delphinus* are very similar, both in the number of transcripts (~250,000) and N50 values (>1400 bp) (Table 3). On the other hand, *M. margaritifera* and *U. crassus* transcriptomes, have a much higher number of assembled transcripts (>1,000,000) and, consequently lower N50 lengths (Table 3). However, all these values are within the reported for other Unionida transcriptomes assembly projects^17–21,23,25–27,29^. Furthermore, *M. margaritifera* and *U. crassus* transcriptome assemblies also have a considerably high level of duplicated BUSCO scores, i.e., around 50%, compared with the remaining species which presented values around 30% (Table 3). The percentage of total genes found (complete + fragmented) in all BUSCO analyses, for all species, was above 95%, except for the *U. pictorum* transcriptome in the Metazoan lineage-specific profile library, which had a total of 93.3%. These results reveal that despite being produced from a single tissue the initial assemblies were highly efficient in capturing conserved and widely express genes, thus providing a highly complete gill transcriptomic repertoire.

**Table 3:** Transrate and Busco scores of redundant and non-redundant gill transcriptome assemblies for each species.

Raw Reads	*Margaritifera margaritifera*	*Unio crassus*	*Unio pictorum*	*Unio mancus*	*Unio delphinus*
Raw sequencing reads	131051306	132002266	104108396	100704688	112439686
Trimmomatic reads removed	1524256 (1.16%)	1761532 (1.33%)	937250 (0.90%)	714904 (0.71%)	1074338 (0.96%)
Centrifuge reads removed	157718 (0.12%)	118410 (0.090%)	101442 (0.097%)	145422 (0.14%)	250936 (0.22%)
Reads used in assembly	129369332 (98.72%)	130122324 (98.56%)	103069704 (99.00%)	99844362 (99.15%)	111114412 (98.82%)

*euk/met. Euk: Dataset with 303 genes of Eukaryota library profile. Met: Dataset with 978 genes of Metazoa library profile.

### Post-assembly processing and annotation verification

The newly assembled transcriptomes were after subject to a decontamination process by Blast-n search against NCBI-nt and Univec databases. The Blast-n hits against NCBI-nt, were manually validated based on the reads with a minimum alignment length of 100 bp, an e-value of 1e-5, an identity score of 90% and a match to Mollusca phylum (NCBI: taxid 6447) or without matches at all, were retained. On the other hand, all Blast-n hits against Univec database were considered exogenous and removed. This decontamination approach has been routinely and successfully used by the team (e.g.^57,58^) and focuses the analyses on the identification, by homology, of putative contaminations and only excluded them if they are well supported and thus avoiding the exclusion of unambiguous matches.

Subsequently, before proceeding to the annotation, the decontaminated transcriptomes were subjected to redundancy removal using Corset. This software relies on hierarchical clustering of contigs that share read alignments and thus allows an unbiased removal of redundancy without discarding non-coding transcripts from the process^45^. The general transcriptome metrics after redundancy removal are presented in Table 3. Corser was extremely efficient in removing the redundancy from the filtered assemblies (Table 3). In fact, over 70% of the initial transcripts were removed during the process, suggesting that although Trinity was effective in producing a complete transcriptome assembly, it as has also generated several duplicated transcripts as well as many transcripts with low read support (Table 3). These results highlight the importance of using read clustering approach to remove redundancy, rather than simply relying on coding transcripts and selection of the largest isoform. The efficiency of the redundancy removal is also supported by the BUSCO analyses, where duplicated scores were on average 3.5% for Eukaryota (n:303) and 2.66% for Metazoa (n:978) after Corset, in opposition to an average 37.32% for Eukaryota (n:303) and 34.96% for Metazoa (n:978) before redundancy removal (Table 3). Furthermore, redundancy removal did not impact the overall completeness of the transcriptome assemblies, which still maintained the total BUSCO scores of over 90% (Table 3). In the end, the final gill transcriptomes were significantly reduced, fairly complete and cleared of putative errors introduced during the assembly, thus properly adjusted for annotation.

TransDecoder prediction of transcripts with an assigned ORF, resulted in a total of 56,730 for *M. margaritifera*, 35,069 for *U. crassus*, 19,830 for *U. pictorum*, 19,881 for *U. mancus*, and 28,216 for *U. delphinus* (Table 4). These predictions were performed in the non-redundant transcriptomes and were deposited in FigShare^56^. Finally, the results of the functional annotation are presented in Table 4, where a thorough listing of hits counts from distinct databases used in the functional annotation processes is presented. The number of transcripts functionally annotated was InterProScan:25,267; Blast:71,046 for *M. margaritifera*, InterProScan:20,432; Blast:51,937 for *U. crassus*, InterProScan:14,723; Blast:24,194 for *U. pictorum*, InterProScan:14,971; Blast:24,775 for *U. mancus* and InterProScan:20,637; Blast:32,688 for *U. delphinus* (Table 4). These values are within the observed values for other Unionida genomics projects, both in transcriptomes^17,19–21,23,25,26^ and genome^14–16,19^. Particularly for *M. margaritifera*, the number of genes functionally annotated, is very similar to the values obtained for the annotated genome assembly available for the species, i.e., 26,836 transcripts^13^.

**Table 4:** Structural and functional annotation statistics for the final gill transcriptome assemblies for each species.

Structural annotation	*Margaritifera margaritifera*	*Unio crassus*	*Unio pictorum*	*Unio mancus*	*Unio delphinus*
Number of transcripts	470852	169668	68670	65620	82542
Number of cdss	56730	35069	19830	19881	28216
Number of exons	56730	35069	19830	19881	28216
Total gene length	442302372	262637793	83762650	89666570	103248722
Total cds length	41461605	34346592	17039142	18840849	22564185
Total exon length	95381543	85666986	36059402	41076667	48847415
mean gene length	939	1547	1219	1366	1250
mean cds length	730	979	859	947	799
mean exon length	1681	2442	1818	2066	1731
**Functional annotation Blast**	***Margaritifera margaritifera***	***Unio crassus***	***Unio pictorum***	***Unio mancus***	***Unio delphinus***
Blast-p/x/n hits (NCBI-RefSeq; NCBI-nr; NCBI-nt)	71046	51937	24194	24775	32688
**Functional annotation InterPro**	***Margaritifera margaritifera***	***Unio crassus***	***Unio pictorum***	***Unio mancus***	***Unio delphinus***
CDD	6295	6475	4357	4693	5542
Coils	4943	4558	2815	2930	3821
GO	10784	9966	7243	7701	10272
Gene3D	15077	13342	9681	9975	13499
Hamap	270	266	221	229	254
InterPro	19126	16611	12116	12524	16717
KEGG	909	874	575	625	802
MetaCyc	835	781	581	574	777
MobiDBLite	10629	8238	5225	5737	6786
PIRSF	628	687	484	556	582
PRINTS	2609	2645	1961	2232	2589
Pfam	15788	14394	10591	11116	14428
ProSitePatterns	3585	3546	2445	2708	3346
ProSiteProfiles	9079	8323	5716	6034	7612
Reactome	3717	3515	2580	2732	3564
SFLD	69	72	54	60	67
SMART	7138	6869	4534	4958	6036
SUPERFAMILY	15070	13240	9376	9729	13190
TIGRFAM	757	751	552	617	815
Total	25267	20432	14723	14971	20637

Overall, these results provide evidence of the quality and completeness of the five gill transcriptome assemblies, which represent timely needed genomic resources for this highly threatened group of organisms. Although future studies should also aim to obtain transcriptomic information from other tissues/development stages, these five annotated gill transcriptomes represent a valuable baseline tool to study these organisms and can ultimately help and guide future conservation actions.

## Data Availability

All software with respective versions and parameters used for producing the resources here presented (i.e., transcriptome assembly, pre and post-assembly processing stages, and transcriptome annotation) are listed in the methods section. Software programs with no parameters associated were used with the default settings.
